# Metachronous colon cancer risk following surgery for first primary rectal cancer in Lynch syndrome

**DOI:** 10.1186/1897-4287-10-S2-A74

**Published:** 2012-04-12

**Authors:** S Parry, AK Win, B Parry, M Kalady, FA Macrae, NM Lindor, RW Haile, PA Newcomb, L Le Marchand, S Gallinger, JL Hopper, MA Jenkins

**Affiliations:** 1 New Zealand Familial Gastrointestinal Cancer Registry, Auckland City Hospital, New Zealand; 2 Department of Gastroenterology, Middlemore Hospital, Auckland, New Zealand; 3 Centre for Molecular, Environmental, Genetic and Analytic Epidemiology, The University of Melbourne, Parkville, Victoria, Australia; 4 Colorectal Surgical Unit, Auckland City Hospital, New Zealand; 5 Department of Colorectal Surgery, Digestive Disease Institute, Cleveland Clinic, Cleveland, Ohio, USA; 6 Colorectal Medicine and Genetics, The Royal Melbourne Hospital, Parkville, Victoria, Australia; 7 Department of Medical Genetics, Mayo Clinic, Rochester, Minnesota, USA; 8 Department of Preventive Medicine, University of Southern California, Los Angeles, California, USA; 9 Cancer Prevention Program, Fred Hutchinson Cancer Research Center, Seattle, Washington, USA; 10 University of Hawaii Cancer Center, Honolulu, Hawaii, USA; 11 Samuel Lunenfeld Research Institute, Mount Sinai Hospital, Toronto, Ontario, Canada; 12 Cancer Care Ontario, Toronto, Ontario, Canada

## Background

It is known that metachronous colorectal cancer risk for Lynch syndrome patients with primary colon cancer is high and total colectomy is the preferred option [1]. However if the index primary cancer is in the rectum, management advice is complicated by considerations of worsening bowel function or stoma formation. To aid surgical decision-making, we estimated the risk of metachronous colon cancer for Lynch syndrome patients who underwent either anterior resection or abdominoperineal resection for primary rectal cancer.

## Methods

This retrospective cohort study comprised 79 MMR gene mutation carriers (18 *MLH1*, 55 *MSH2*, 4 *MSH6* and 2 *PMS2*) from the Colon Cancer Family Registry who had a surgical resection for their first primary rectal cancer. Age-dependent cumulative risks of metachronous colon cancer were calculated using the Kaplan-Meier method. Risk factors for metachronous colon cancer were assessed using a Cox proportional hazards regression.

## Results

During 866 person-years of observation (median 9 years; range 1-32 years) since diagnosis of first rectal cancer, a total of 21 (27%) carriers were diagnosed with metachronous colon cancer (incidence 24.2; 95% CI 15.8–37.2 per 1000 person-years). Incidence for carriers who had an anterior resection (26.8; 95% CI 15.5–46.1 per 1000 person-years) was not different from that for carriers who had an abdominoperineal resection (21.0; 95% CI 10.5–42.1 per 1000 person-years) (*P*=0.1). Cumulative risk of metachronous colon cancer was 19% (95% CI 9–31%) at 10 years, 47% (95% CI 31–68%) at 20 years and 69% (95% CI 45–89%) at 30 years after surgical resection. There was no difference in the frequency of surveillance colonoscopy between two types of surgery (one colonoscopy per 1.1 (95% CI 0.9–1.2) years after anterior resection vs. one colonoscopy per 1.4 (95% CI 1.0–1.8) years after abdominoperineal resection).

## Conclusions

For carriers of MMR gene mutations who contract rectal cancer, the metachronous cancer risk is substantial and mirrors that seen for carriers undergoing segmental resection for primary colon cancer [1], despite the majority continuing to receive frequent surveillance colonoscopy. This risk needs to be considered when the extent of surgery for primary rectal cancer is planned. Whereas total colectomy for primary colon cancer in mutation carriers is appropriate, for primary rectal cases this strategy has major implications for continence and need for stoma. Nevertheless, given the high metachronous risk, this needs serious consideration especially for younger patients.
